# Occupational Therapy in the Intensive Care Unit: A Retrospective Cohort Study

**DOI:** 10.1155/oti/4043807

**Published:** 2026-05-18

**Authors:** So Hyun Jeon, Sung Eun Hyun, Chae Hyeon Lee, Yeong Ae Yang

**Affiliations:** ^1^ Department of Rehabilitation Medicine, Seoul National University Hospital, Seoul, Republic of Korea, snuh.org; ^2^ Department of Occupational Therapy, Inje University, Gimhae-si, Gyeongsangnam-do, Republic of Korea, inje.ac.kr; ^3^ Department of Rehabilitation Medicine, Seoul National University College of Medicine, Seoul, Republic of Korea, snu.ac.kr

**Keywords:** critical care, critical illness, intensive care unit, occupational therapy, rehabilitation

## Abstract

**Importance:**

Determining whether individualized occupational therapy (OT) can be feasibly and safely provided to intensive care unit (ICU) patients is essential.

**Objective:**

The aim of this study is to investigate the feasibility and safety of providing individualized OT programs in the ICU based on functional assessment outcomes.

**Design:**

This study was designed as a retrospective cohort study.

**Setting:**

The study was conducted in the ICU of a tertiary university hospital.

**Participants:**

Patients referred for ICU rehabilitation who met predefined ICU OT inclusion and exclusion criteria and initiated ICU OT between February 2021 and December 2023 were included.

**Intervention:**

Based on functional evaluation, each patient′s individualized OT program was classified into cognitive training, functional and activities of daily living (ADLs) training, or a combination of cognitive training and functional and ADL training.

**Outcomes and Measures:**

Feasibility was assessed based on eligibility, initiation and interruption rates, time to OT initiation, missed or deferred sessions, and adherence to the intervention framework. Safety was evaluated by whether OT sessions were discontinued or prescriptions were terminated owing to adverse events based on predefined criteria during OT delivery.

**Results:**

Among 828 patients referred for ICU rehabilitation, 184 (22%) met eligibility criteria, and 158 patients initiated the ICU OT program. Among these, 55 patients (34.8%) completed all prescribed OT sessions, whereas 103 (65.2%) did not, yielding a program interruption rate of 65.2%. The median time from OT prescription to the first session was 1 day. No sessions were canceled owing to patient refusal, and only one session (0.15%) was deferred because of agitation. Only one adverse event (hypotension) occurred during OT delivery.

**Conclusion and Relevance:**

Individualized ICU OT can be feasible and safe to be implemented for ICU patients who meet predefined eligibility criteria when interventions are guided by structured functional assessments and clearly defined criteria for treatment discontinuation or termination.

## 1. Introduction

Occupational therapy (OT) in the intensive care unit (ICU) has garnered increasing focus owing to its role in enhancing functional outcomes [1–3]. Approximately 30%–80% of ICU patients develop persistent sequelae even after discharge, known as postintensive care syndrome (PICS), comprising physical, cognitive, and psychological impairments. Patients with PICS have difficulties in attention, memory, and executive function, significantly limiting activities of daily living (ADLs). OT intervention can mitigate these impairments by providing targeted cognitive rehabilitation and training in ADLs, thereby helping in improving functional outcomes and quality of life for ICU survivors [4–6].

However, coherent OT provision in current ICUs remains globally insufficient [7, 8]. A US survey indicated that only 35% of Americans have access to OT and physical therapy in ICUs [9]. Most ICU rehabilitation roles focus on mobility and physical rehabilitation rather than cognition [10–14]. To the best of our knowledge, no study has systematically reported OT program application for ICU patients in terms of patient referral, treatment selection according to functional level, and same‐day adjustments based on arousal state [8, 10]. Before evaluating occupation‐focused outcomes, determining whether OT interventions can be consistently initiated, delivered, and maintained in the ICU environment, where medical instability and system constraints often limit rehabilitation delivery, is essential. Accordingly, we aimed to assess the feasibility and safety of delivering an individualized OT program based on functional assessments in critically ill patients.

## 2. Materials and Methods

### 2.1. Ethics Statements

This study was approved by the Seoul National University Hospital Clinical Research Ethics Center on January 25, 2023 (IRB Approval Number 2301‐090‐1395) and was conducted in accordance with the Declaration of Helsinki. The requirement for participants′ informed consent was waived owing to the retrospective nature of the study.

### 2.2. Study Design and Participants

This observational retrospective cohort study included patients admitted to the ICU and prescribed ICU OT between February 2021 and December 2023 in a 1761‐bed tertiary hospital with five types of ICUs (medical ICU, surgical ICU, semi‐ICU, emergency ICU, and cardiopulmonary ICU) equipped with 106 beds. Upon ICU admission, all patients who were anticipated to recover from critical illness had been referred to the Department of Rehabilitation Medicine for ICU rehabilitation within 48 h (preferably within 36 h). In monthly team meetings, ICU attending physicians and ICU rehabilitation therapists discussed the necessity of a structured ICU rehabilitation program, leading to the establishment of the ICU OT programs [8, 10, 15] based on each patient′s clinical needs. They also defined inclusion and exclusion criteria (Supporting Information 1) for the selection of candidates who could receive additional ICU OT with conventional physical therapy.

The inclusion criteria required the ability to follow simple commands, manageable delirium, and hemodynamic and respiratory stability. The exclusion criteria included hypotension, tachycardia, progressive respiratory failure, progressive respiratory failure, acute medical deterioration, and planned procedures or sedation. “Exclusion” referred to cases in which OT was not prescribed because the patient met the exclusion criteria for the ICU OT program. After OT was initiated, therapists monitored clinical warning signs corresponding to the exclusion criteria before and during each session to determine whether they can proceed with the treatment. These clinical signs included hemodynamic instability, ventilator dyssynchrony, respiratory instability, subjective pain, and physical resistance during the session, and when such signs were observed, the session was immediately discontinued. “Discontinuation” referred to situations in which the OT prescription was retained, but the session was not performed owing to safety concerns. If detailed reassessment after session discontinuation showed that the patient continued to meet the exclusion criteria, the OT prescription was terminated. “Termination” referred to cases in which the entire prescribed OT program could no longer be provided and was therefore stopped. OT sessions that were discontinued or led to termination of the OT program because predefined discontinuation criteria were met during treatment were documented and classified as adverse events. “Ending” was used as a comprehensive term indicating that the prescribed ICU OT program was concluded for any reason, including cases in which the prescribed intervention was fully completed and those in which the program was concluded due to interruption. “Interruption” was defined as failure to complete the planned five‐session ICU OT program after initiation, including early ending due to transfer to the general ward, discharge, clinical improvement leading to discontinuation of OT, medical decompensation, or surgery.

### 2.3. Feasibility and Safety

The feasibility of the ICU OT program was determined by evaluating the proportion of patients meeting the eligibility criteria for the program among those referred for ICU rehabilitation and the program interruption rate, time to initiation of OT after prescription, missed or deferred sessions and their reasons, and adherence to the planned ICU OT program. The program interruption rate was defined as failure to complete the planned five‐session ICU OT program. In this study, safety referred to the safety of delivering OT in the ICU. Safety was assessed as the proportion of patients for whom the ICU OT program was discontinued or terminated owing to a safety event, classified according to predefined exclusion criteria for the ICU OT program (with detailed descriptions provided in Supporting Information 1). Additionally, the duration of ICU OT (days) referred to the actual number of days of ICU OT, calculated as calendar days from the first OT evaluation to the final OT session performed in the ICU.

### 2.4. Intervention

#### 2.4.1. Therapist Qualifications

One occupational therapist (> 7 years of experience) was assigned exclusively to ICU OT and treated an average of eight patients for 4 h each day. In addition to extensive clinical experience, the therapist had completed comprehensive hospital‐based training and practical instruction in essential ICU competencies, including critical care monitoring, recognition of physiological instability, application of eligibility criteria, management of critically ill patients, and standardized administration of all assessment tools used in this study. The therapist also participated regularly in monthly educational sessions and case conferences with the dedicated ICU rehabilitation team, thereby continuously updating their knowledge of current ICU rehabilitation guidelines and protocols.

#### 2.4.2. Functional Assessment

All referred OT patients were assessed and recorded during the first treatment session by a designated occupational therapist in charge of ICU rehabilitation. Functional assessments were conducted using the Manual Muscle Test (MMT) for limb strength [16], the Korean Mini‐Mental State Examination‐2nd Edition (K‐MMSE2) for cognitive function [17, 18], the Modified Barthel Index (MBI) to assess the level of ADLs [19], and handgrip strength (HGS) to measure muscle strength using a hand dynamometer [20]. Each evaluation, along with its cutoff score, is commonly used in the clinical ICU setting to assess cognitive and physical function [7, 21, 22]. The pointing version of the K‐MMSE2 was used for nonverbal patients. The use of the K‐MMSE2 in this study was approved by the copyright holder. The corresponding permission document is included as Supporting Information 2.

**Figure 1:**
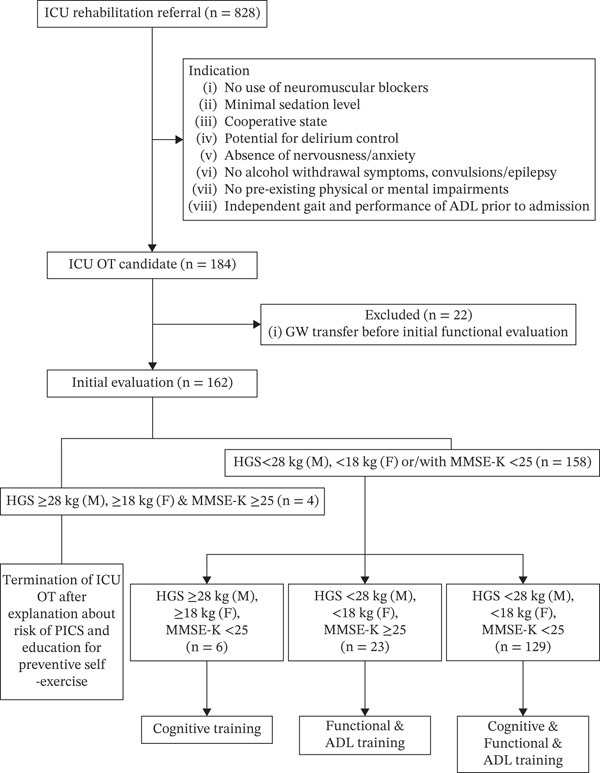
Flowchart of the study. Abbreviations: ICU, intensive care unit; OT, occupational therapy; ADLs, activities of daily living; GW, general ward; HGS, handgrip strength; K‐MMSE2, Korea Mini‐Mental State Examination; PICS, postintensive care syndrome; SBP, systolic blood pressure; DBP, diastolic blood pressure.

Cognitive intervention was included in the ICU OT program for patients with a K‐MMSE2 score of 24 or less, which is the cutoff score for defining cognitive decline [17, 18]. HGS was measured using a Jamar dynamometer (Model 5030 J1, Sammons Preston Rolyan, Bolingbrook, IL, United States). Low muscle strength was defined as HGS < 28 kg for men and < 18 kg for women, following the clinical cutoff scores for sarcopenia (Figure 1) [23, 24].

**Figure 2:**
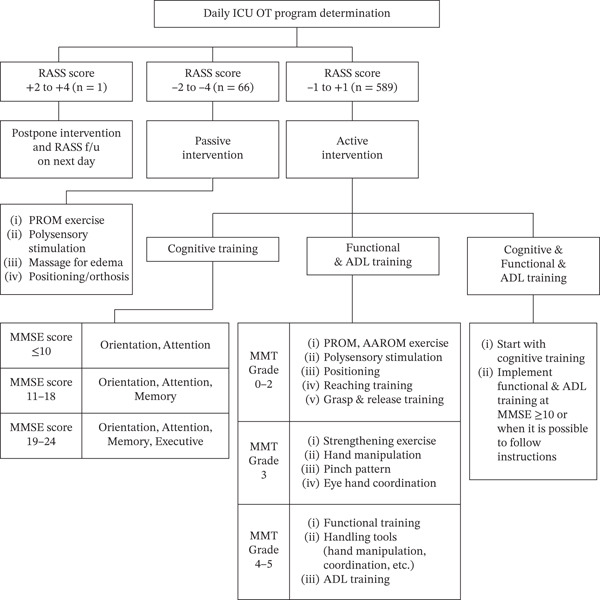
Daily adjustment of ICU OT according to the patient′s state of agitation–sedation on the day.

The Richmond Agitation–Sedation Scale (RASS) score was also assessed before the start of each treatment session and recorded daily. The results were used to assess changes in patients′ medical condition and determine and/or change the contents and goals of the daily ICU OT program using an algorithm described in Figure 2 [25, 26].

We also used the MBI as a supplementary assessment, which is a measure of functional independence assessing 10 items of ADLs, to determine the type and contents of ADL training for each patient but not to determine whether ICU OT should be provided. This is because the MBI cannot accurately represent actual ADL status in the ICU setting because most patients are connected to various medical devices, often requiring assistance regardless of their functional capacity [27]. Detailed explanations for each assessment tool are also summarized in Supporting Information 3.

Despite the availability of internationally validated ICU‐specific functional assessment tools, most tools focus on mobility and physical performance rather than OT‐related outcomes. Furthermore, no widely applicable ICU‐specific outcome measure tailored to OT is currently available [28]. Notably, a Cochrane systematic review reported that ICU rehabilitation studies predominantly evaluated outcomes related to physical function, muscle strength, and mobility, with considerable heterogeneity in the outcome measures used across studies [29]. Commonly used assessment tools include the Medical Research Council sum score for muscle strength and measures of physical function and mobility, such as the Physical Function in ICU Test, Acute Care Index of Function, Functional Status Score for the ICU (FSS‐ICU), and ICU Mobility Scale, as well as ADL measures such as the Barthel index [28, 29]. Considering these limitations, the assessment tools and corresponding cutoff values used in this study were selected based on their established validity and clinical utility in the literature. These measures are widely used as standard clinical approaches for comprehensively evaluating cognitive and physical function across acute care settings, including the ICU [20, 23, 24]. Accordingly, we applied standardized clinical criteria to systematically assess the functional status of ICU patients and to guide individualized OT intervention planning.

#### 2.4.3. ICU OT Program

Based on the initial functional assessment, an individualized ICU OT program was prescribed, with each OT session structured as a 30‐min intervention delivered once daily for a total of five sessions. A structured description of the ICU OT intervention based on the Template for Intervention Description and Replication framework is provided in Table 1. The content of each session was individually tailored to the patient′s cognitive status, physical function, and level of activity performance. If the HGS and K‐MMSE2 scores were above the threshold, no additional OT intervention was deemed necessary. In this case, only an explanation of the risk of PICS and education on preventive self‐exercise were provided (classified as “no impairment to continue ICU OT”). If only the K‐MMSE2 score was below the threshold, cognitive training was provided. If only the HGS was below the threshold, functional and ADL training were conducted. If both HGS and K‐MMSE2 scores were below the threshold, combined cognitive, functional, and ADL training were provided (Figure 1).

**Table 1:** Structured description of the ICU OT intervention according to the Template for Intervention Description and Replication (TIDieR) framework.

Domain (TIDieR)	Description
Intervention name	Individualized ICU OT program.
Rationale/theory	The ICU OT program was developed to assess patients′ functional status using standardized assessments and to determine and apply intervention types based on assessment results. This study was aimed at assessing the feasibility and safety of delivering an individualized OT program in the ICU.
Target population	Patients admitted to the ICU (medical ICU, surgical ICU, semi‐ICU, emergency ICU, and cardiopulmonary ICU) and referred to the Department of Rehabilitation Medicine for ICU rehabilitation were included. Referrals were made within 48 h of ICU admission (preferably within 36 h). Inclusion criteria included the ability to follow simple commands under light sedation (not induced by neuromuscular blockers), manageable delirium without severe agitation, and hemodynamic and respiratory stability. Active ICU OT was recommended for patients with functional impairments requiring rehabilitation and without a planned early transfer to the general ward. Exclusion criteria included physiological instability such as hypotension, severe bradycardia or tachycardia, progressive respiratory failure, oxygen desaturation, acute medical deterioration, increased intracranial pressure, overt gastrointestinal bleeding, acute myocardial infarction, planned medical or surgical procedures, or agitation requiring sedation. Eligibility for ICU OT was determined based on these inclusion and exclusion criteria to ensure safe participation.
Provider	ICU OT was prescribed by a physician in the Department of Rehabilitation Medicine. The ICU OT intervention was delivered by one occupational therapist assigned exclusively to the ICU OT. The occupational therapist had more than 7 years of clinical experience and had completed hospital‐based training in ICU rehabilitation.
Setting	All interventions were performed at the patient′s bedside in the ICU.
Materials/equipment	Portable and nonhazardous materials were used in the ICU setting. Cognitive training used paper‐based materials, puzzles, softballs, and tablet computers. Upper limb functional training used handgrip devices, weight training tools, resistance bands, and pegboards. ADL training used utensils (chopsticks, spoons, and forks), plastic bottles, paper cups, gloves, masks, ribbon, and buttoning tools. Passive intervention used positioning materials and orthoses.
Core components	The ICU OT program consisted of cognitive training; upper limb functional training; ADL training; combined cognitive, functional, and ADL training; passive intervention; and education only (no impairment group). Cognitive training included orientation, attention, memory, and executive function training. Upper limb functional training included range of motion exercise, strengthening exercise, reaching, grasping, manipulation, and coordination training. ADL training included hygiene, feeding, and dressing activities. Passive intervention included polysensory stimulation, edema massage, passive range of motion exercises, positioning, and orthoses. Intervention type was determined based on K‐MMSE2, HGS, MMT, MBI, and RASS assessment results.
Dose (intensity)	Each ICU OT session was structured as a 30‐min intervention.
Frequency	ICU OT was delivered once daily.
Duration	The ICU OT program consisted of a total of five sessions.
Progression criteria	Intervention progression was determined based on K‐MMSE2, HGS, MMT, MBI, and RASS assessment results. Cognitive training progression was applied according to K‐MMSE2 score ranges (≤ 10, 11–18, and 19–24). Functional training progression was applied according to MMT grades (0–2, 3, and 4–5). Intervention type selection was based on HGS cutoff (< 28 kg for men and < 18 kg for women), K‐MMSE2 cutoff (≤ 24), and RASS level (−4 to +4). Active intervention was performed for patients with RASS scores of −1 to +1, passive intervention for patients with RASS scores of −2 to −4, and intervention was deferred for patients with RASS scores of +2 or higher.
Tailoring/adaptation	Intervention selection was determined based on cognitive function, muscle strength, ADL performance, and arousal level.
Safety monitoring	Therapists continuously monitored patients for clinical warning signs based on the inclusion and exclusion criteria before and during each session. Clinical warning signs included hemodynamic instability, ventilator dyssynchrony, respiratory instability, subjective pain, and physical resistance. If clinical warning signs were observed, the session was immediately discontinued, and physiological parameters corresponding to the exclusion criteria were promptly reassessed. If the reassessment continued to meet the exclusion criteria for the ICU OT program, the OT prescription was terminated. All discontinued sessions and terminations were documented and classified as adverse events.

Figure 2 describes the daily adjusted ICU OT program individualized for each patient. An active intervention was performed for patients with RASS scores of −1, 0, or +1 following a prescription of either cognitive or functional/ADL training or both. Passive intervention was performed for patients with RASS scores of −2, −3, or −4, comprising polysensory stimulation, edema massage, passive range of motion (PROM) exercises, positioning, and orthoses when necessary. Interventions for patients with RASS scores of +2, +3, or +4 were deferred to the next day because the patients were unable to follow the therapist′s instructions.

Cognitive training was performed using low‐scoring K‐MMSE2 items (e.g., orientation, attention, memory, and problem‐solving). Patients with K‐MMSE2 scores of 10 or less received orientation and attention‐based treatment. For patients with K‐MMSE2 scores of 11–18 who underwent the previously mentioned treatment, interventions related to memory training were added. For patients with scores of 19–24 and those who had undergone the previous training, further interventions for executive function improvement were included. Traditional cognitive interventions were performed in the ICU setting using nonhazardous and portable tools, for example, paper, softballs, puzzles, and sometimes tablet computers.

Upper limb functional training was recommended for those with HGS of < 28 kg in men and < 18 kg in women. The details of the treatment were determined based on each patient′s generalized muscle strength of the upper limb, as assessed using the MMT. This is a 6‐grade scale, where Grade 0 refers to *a state of no contraction or muscle movement*, Grade 1 refers to *a state in which muscles contract but do not move*, Grade 2 refers to *a state in which only horizontal movement is possible*, Grade 3 refers to *a movement against gravity but not resisting additional resistance*, Grade 4 refers to *a movement against external resistance with weaker intensity than usual*, and Grade 5 refers to *a movement with normal intensity* [30]. For MMT Grades of 0–2, ICU OT comprised polysensory stimulation, positioning, PROM, and active‐assistive range of motion training, as well as reaching, grasping, and release training. For MMT Grade 3, strengthening exercise, hand manipulation, pinch pattern, and eye–hand coordination training (with tools) followed. Patients with MMT Grades 4–5 underwent mostly training in functional training, handling tools (such as hand manipulation and coordination), and further high‐level ADL training. Safe and portable tools, such as handgrips and weight training tools, resistance bands, and various pegboards, were used for treatment.

ADL training started with the simplest and basic activities, and individualized OT sessions focused on the domain evaluated as most deficient among the 10 ranked as most necessary in ADL training, according to initial MBI results. Basic ADLs were either simulated or actually executed in either a supine and long‐sitting or short‐sitting position, encompassing hygiene (e.g., washing hands, wiping the face, donning and doffing masks, and simulated tooth brushing), feeding (e.g., utilizing chopsticks, spoons, and forks and pouring water from a plastic bottle into a paper cup), and dressing (e.g., putting on gloves, removing gloves, tying a ribbon, and buttoning activities). Treatment plans, fundamentally protocol‐based, were tailored to each patient′s unique needs. Therapists aimed to optimize outcomes by considering patients′ conditions, preferences, and environment, thereby enhancing satisfaction and treatment outcomes.

### 2.5. Permission for the Use of MMSE

The assessment tool used in this study was K‐MMSE2. This tool was purchased through the official website, and a license to use for research purposes (Korean version) was permitted by INPSYT Co. Ltd. (source of the MMSE version used: https://inpsyt.co.kr/main).

### 2.6. Statistical Analysis

Clinical data collected during the treatment process were retrospectively analyzed. The electronic medical records, including ICU rehabilitation and medical conditions, were thoroughly reviewed manually to verify the information. Frequency analysis was conducted for participant characteristics using descriptive statistics. Categorical variables are expressed as frequencies and percentages, and continuous variables are expressed as means with standard deviations (SDs) or medians with interquartile ranges (IQRs).

## 3. Results

### 3.1. Patient Characteristics

The demographics and clinical characteristics of 162 participants are summarized in Table 2. The study population consisted of 52 patients (32.1%) with respiratory diseases, including pneumonia and acute respiratory distress syndrome; 48 patients (29.6%) with cardiovascular diseases, including heart failure and myocardial infarction; 28 patients (17.3%) with digestive diseases, such as liver cancer and liver cirrhosis; 21 patients (13.0%) with neurological diseases, including stroke and brain tumors; and 13 patients (8.0%) with other severe medical conditions, including acute kidney injury and acute myeloid leukemia. The semi‐ICU is a bridging ward for patients transferred from the ICU before being transferred to the general ward and is specialized for patients who are in the recovery phase but still need intensive monitoring. Three participants from the semi‐ICU began ICU OT after being transferred from the medical ICU (*n* = 2) or surgical ICU (*n* = 1) because of rapid recovery and transfer (Table 2).

**Table 2:** Demographic characteristics of the included patients (*N* = 162).

Variable	Patients in the ICU
Age (years)	64.20 (15.06)
Male/female gender	96 (59.3%)/66 (40.7%)
BMI (kg/m^2^)	23.37 (6.02)

ICU type	
MICU	72 (44.4%)
SICU	56 (34.6%)
CPICU	25 (15.4%)
EICU	6 (3.7%)
Semi‐ICU	3 (1.9%)

ICU admission category	
Respiratory	52 (32.1%)
Cardiovascular	48 (29.6%)
Digestive	28 (17.3%)
Neurological	21 (13.0%)
Others	13 (8.0%)
Length of intubation (days)	28.10 (43.26)
Length of ICU stay (days)	16.82 (19.35)
Length of hospital stay (days)	83.09 (70.45)
Tracheostomy	76 (46.9%)

Discharge status	
Discharged to home	73 (45.1%)
Deceased	49 (30.2%)
Transferred to another hospital	40 (24.7%)

*Note:* Data are expressed as *n* (percentage) or mean (standard deviation).

Abbreviations: BMI, body mass index; CPICU, cardiopulmonary intensive care unit; EICU, emergency intensive care unit; ICU, intensive care unit; MICU, medical intensive care unit; semi‐ICU, semi‐intensive care unit; SICU, surgical intensive care unit.

### 3.2. Feasibility

Among the 828 patients referred for ICU rehabilitation, 184 patients (22%) were identified as eligible for the ICU OT program according to the predefined inclusion and exclusion criteria (Figure 1; detailed criteria are summarized in Supporting Information 1). Of these, 22 patients were transferred to the general ward before the initial OT evaluation, and four were classified as having no impairment requiring continued ICU OT. Consequently, 158 patients initiated the ICU OT program and underwent the initial evaluation (Figure 1). Among these patients, 55 (34.8%) completed all prescribed OT sessions; the program interruption rate was 65.2% (103 patients). The primary reasons for interruption included transfer to the general ward (93, 58.9%), discharge (2, 1.3%), rapid clinical improvement leading to discontinuation of OT (2, 1.3%), medical decompensation precluding continuation of OT (5, 3.2%), and surgery (1, 0.6%).

The median time from OT prescription to the first session was 1 day, as OT was initiated on the next working day after prescription (excluding weekends and public holidays). The initial OT evaluation was initiated on the same day that ICU OT was prescribed by the rehabilitation physician. No ICU OT sessions were canceled owing to patient refusal, and one session (0.15%) was deferred because of agitation. All interventions were delivered according to the predefined ICU OT intervention framework, with daily adjustments according to RASS and functional criteria.

On initial evaluation, in male patients, the average strength of dominant hands was 10.12 kg (SD = 8.63), and that of nondominant hands was 9.24 kg (SD = 8.60). In female patients, the average strength of dominant hands was 7.10 kg (SD = 5.53), and that of nondominant hands was 5.39 kg (SD = 5.54). Additionally, the mean K‐MMSE2 and MBI scores were 15 (SD = 9.29) and 6.26 (SD = 6.81), respectively. Based on initial evaluation results, six (3.8%) patients needed cognitive training alone, 23 (14.6%) needed functional/ADL training, and 129 (81.6%) required intensive ICU OT, including combined cognitive, functional, and ADL training (Table 3). The results of the initial OT evaluation of 158 people are presented in Table 3.

**Table 3:** Occupational therapy interventions in the ICU (*N* = 158).

Variable	Value
Type of treatment required based on initial evaluation	
Cognitive training	6 (3.8%)
Functional and ADL training	23 (14.6%)
Cognitive, functional, and ADL training	129 (81.6%)
Number of ICU OT sessions per patient	4 (1–7)
Duration of ICU OT (days)	4.88 ± 1.61
Adverse events^a^	1 (0.6%)

Reasons for ending	
Completion of prescribed treatments	55 (34.8%)
Transferred to the general ward	93 (58.9%)
Discharged	2 (1.3%)
Improved quickly enough to discontinue OT	2 (1.3%)
Medical decompensation to continue OT	5 (3.2%)
Operation	1 (0.6%)
Continuation of OT after being transferred to the general ward	47 (29.7%)

*Note:* The duration of ICU OT (days) was defined as the number of calendar days during which OT was actually delivered in the ICU, calculated from the initial ICU OT evaluation to the final ICU OT session.

Abbreviations: ADLs, activities of daily living; ICU, intensive care unit; OT, occupational therapy.

^a^Adverse events were defined as the occurrence of clinical warning signs or medical conditions, including hemodynamic instability, ventilator incompatibility, unstable breathing, verbal or nonverbal pain responses, physical resistance, new arrhythmias or electrocardiographic changes, increased intracranial pressure, overt gastrointestinal bleeding, or acute myocardial infarction that resulted in immediate discontinuation of the ICU OT session and/or termination of the OT prescription.

### 3.3. Safety

According to the RASS criteria, most ICU OT sessions were completed with active intervention (n = 589), whereas 66 sessions required passive intervention owing to the patient being in a sedated state; one session was postponed because of irritability (Figure 2). All 158 patients were able to participate in treatment and received a total of 656 ICU OT sessions. The median number of completed ICU OT sessions per patient was 4 (IQR 1–7) among the five prescribed sessions, and the duration of OT was 4.88 days (SD 1.61). Reasons for ending the ICU OT program included completion of all prescribed OT sessions in 55 patients (34.8%), transfer to the general ward in 93 patients (58.9%), hospital discharge in two patients (1.3%), sufficient clinical improvement to discontinue OT in two patients (1.3%), medical decompensation preventing further OT in five patients (3.2%), and planned surgery in one patient (0.6%).

Five patients terminated OT owing to a decline in medical status. Among them, in four patients, this was due to underlying disease progression, and in one patient (0.6% of patients who received ICU OT), OT was initially discontinued after an adverse event occurred during treatment and was subsequently terminated. This one patient experienced occasional orthostatic hypotension, owing to a history of tension pneumothorax and chylothorax, whenever they tried to sit for OT intervention. These events were not induced by OT but were due to underlying medical instability. During the third treatment session, the patient′s blood pressure dropped below the exclusion thresholds (SBP < 90 mmHg and DBP < 50 mmHg) on more than two occasions, and ICU OT was therefore discontinued early following reassessment after termination of the prescription (Table 3). In this patient, these episodes were classified as non‐OT‐related adverse events based on clinical reassessment by the treating ICU physician.

## 4. Discussion

This study reports that individualized OT can be feasibly and safely used in ICU patients when the goals and content of the treatment program are individualized based on assessments of cognitive and physical functions, activity levels, and arousal status. Initial results indicated that a significant proportion of patients required comprehensive OT programs and that cognitive, upper limb, and ADL interventions were delivered according to the predefined program. Therefore, the ICU OT program can be consistently delivered for ongoing assessment and intervention in patients with daily fluctuating conditions and diverse diagnoses in different ICU settings.

Among the 828 patients referred for ICU rehabilitation, only 184 (22.2%) met the eligibility criteria for ICU OT. This low eligibility rate can be attributed to two primary factors. First, early ICU OT referrals depend heavily on the clinicians′ judgment at the time of ICU admission, which may have led to inappropriate referrals of patients who did not truly meet the criteria. Second, many patients were medically unstable at the time of referral and were excluded based on strict eligibility criteria. Consequently, both patient instability and the rigor of the exclusion standards substantially limited the number of individuals who could safely participate in treatment. Furthermore, in this study, none of the ICU OT sessions delivered to the eligible patients were canceled owing to patient refusal. This is likely because patients with a high likelihood of refusing therapy, such as those experiencing severe pain, fatigue, anxiety, or poor cooperation, had already been excluded during the initial eligibility assessment. Nonetheless, prioritizing patient safety over expanding the number of treated patients is essential, and the eligibility criteria applied in this study are considered appropriate. Similar trends have been observed in previous research; for example, Deemer et al. [31] reported that only 70 of 408 screened patients (17%) were eligible. This suggests that the pool of ICU patients eligible for OT is inherently limited, and the eligibility rate in our study is consistent with international evidence. Consequently, the patients who ultimately received ICU OT were medically stable and able to cooperate, and the treatment sessions were flexibly adjusted according to each patient′s clinical status. These factors collectively minimized the likelihood of treatment refusal, enabling all scheduled sessions to be conducted as planned. In the same study, only a small proportion of sessions (eight of 110, 7%) were terminated early at the patient′s request, indicating that treatment refusal is a relatively infrequent occurrence. In addition, the low eligibility rate highlights directions for program refinement. Enhancing ICU staff education on OT‐specific referral criteria can reduce unnecessary referrals and facilitate more accurate identification of appropriate candidates. Introducing a simple assessment checklist based on inclusion and exclusion criteria, developed in collaboration with ICU clinicians, may help identify suitable patients at an earlier stage. Furthermore, establishing a routine re‐evaluation process can enable the timely initiation of OT for patients whose clinical status improves. These refinements may contribute to improving the accessibility of the ICU OT program.

Continuous monitoring of inclusion and exclusion criteria was maintained throughout the ICU OT program. Based on this monitoring, four patients terminated ICU OT owing to progression of their underlying medical conditions, and one patient experienced transient orthostatic hypotension during an ICU OT session while transitioning to a seated position, resulting in immediate session discontinuation in accordance with the predefined discontinuation criteria. Two transient episodes occurred during the same session, during which the patient′s blood pressure temporarily fell below the exclusion thresholds. Both episodes resolved promptly with postural adjustment and required no additional medical intervention. Following session discontinuation, a detailed medical reassessment was conducted, which confirmed that the patient continued to meet the exclusion criteria. Based on clinical evaluation by the treating physician, these events were attributed to the patient′s pre‐existing pleural pathology and underlying medical conditions rather than to the OT intervention itself. Accordingly, the OT prescription was subsequently terminated in accordance with the safety protocol. These events did not affect the patient′s overall medical course, supporting that ICU OT can be safely delivered when conservative eligibility standards are applied. Moreover, physiological abnormalities frequently occur in the ICU independent of rehabilitation activities. These observations are consistent with the established safety profile of ICU rehabilitation. Recent systematic reviews report that hemodynamic fluctuations, such as blood pressure instability, occur at approximately 3.8 events per 1000 sessions but rarely impact patient management while simultaneously demonstrating a low rate of potential safety events (2.6%) and rare occurrences requiring medical intervention [32]. These findings suggest that strict eligibility criteria and stepwise reassessment procedures play an important role in supporting the continued delivery of ICU OT and enable safe program implementation in actual clinical settings.

Most patients exhibited HGS and K‐MMSE2 scores below the criteria and RASS scores between −1 and +1, indicating eligibility for active intervention, which included cognitive, functional, and ADL training. Although ICU OT is increasingly recognized, most recent studies on ICU rehabilitation continue to be focused mainly on mobility and physical treatment rather than on cognitive or upper limb functional training [10–14]. Therefore, the implementation of cognitive and upper limb OT interventions in the ICU remains limited in current rehabilitation practice. Our findings revealed that only 14.6% of patients required upper extremity functional treatment and 3.8% required cognitive treatment alone. Notably, 81.6% required a combined regimen including upper extremity function, cognitive, and ADL training, based on baseline functional assessments. These findings describe the functional profiles identified at the initial OT evaluation and may provide reference information for designing individualized ICU OT programs incorporating cognitive, upper extremity, and ADL components. As this study was not designed to evaluate treatment effectiveness, further prospective studies are warranted to determine the impact of ICU OT on functional outcomes. Thus, this study should be interpreted as a necessary preliminary step, as establishing feasibility is important before conducting outcome‐driven trials focusing on occupation‐based recovery.

A total of 158 (85.9%) eligible patients initiated ICU OT, and owing to limited therapist availability, each patient was prescribed five sessions. Among them, 55 (34.8%) completed all prescribed sessions. Of the 93 (58.9%) patients whose treatment ended owing to transfer to a general ward, 28 (29.7%) continued therapy after transfer, suggesting additional rehabilitation needs beyond the ICU‐based intervention. These quantitative constraints are consistent with recent systematic reviews, which highlight the persistent lack of evidence regarding the optimal rehabilitation dose in the ICU and attribute this gap largely to structural limitations [2]. International studies have similarly identified staffing shortages as an ongoing obstacle to providing adequate ICU rehabilitation [9, 33, 34]. The limited therapy dose resulting from staffing constraints may have limited the scope of ICU OT delivery for some patients. In this study, the number of sessions prescribed to patients was influenced by staffing and operational conditions in the ICU rather than being based solely on patients′ functional requirements. Therefore, the prescribed intervention dose may not have been sufficient, and this should be taken into account when interpreting the feasibility results and program interruption rate. With future expansion of personnel and resources, the operational structure of ICU OT delivery may be improved, enabling more stable implementation of ICU OT programs.

In this study, missed sessions occurred across the prescribed intervention sessions owing to various factors, which increased the program interruption rate and directly influenced the feasibility assessment. These missed sessions did not occur randomly but resulted from predefined criteria based on factors inherent to the ICU environment, such as patients′ clinical instability, transfer to the general ward, and limited human resources. Therefore, the relatively high program interruption rate should be interpreted not solely as a limitation of the intervention itself but rather as reflecting the inherent characteristics of real‐world ICU care, including early transfer and resource constraints. In addition, we defined completion of the intervention as the performance of all planned sessions. However, given the nature of the ICU setting, completion does not necessarily indicate clinical effectiveness or treatment success. Because interventions are dynamically adjusted according to changes in patient condition, early transfer or discharge, and resource availability, completion should be interpreted not as an absolute outcome measure but as an indicator of the program implementation process.

Some previous studies separately reported conducting the arousal level assessment and early functional assessment (HGS and K‐MMSE2), but no previous study has reported conducting these two domains of assessments to optimize individualized ICU OT programs [35–37]. Critically ill patients exhibit daily fluctuations in arousal levels owing to sedation status or impaired consciousness [38]; therefore, assessing their current level of arousal before each treatment session is needed. In this study, the RASS was used at every session to evaluate arousal status, and the scope and direction of interventions were adjusted accordingly. These initial and session‐by‐session assessments of arousal were conducted to provide individualized ICU OT for patients whose clinical condition often fluctuates. In future studies, if structured intervention strategies are developed based on a patient′s arousal level over a sufficient period of time, OT could also be adjusted and provided to patients in an agitated state to manage delirium [39, 40]. Depending on the resources and clinical context of each institution, assessments of delirium (e.g., the Confusion Assessment Method for the ICU) and depressive symptoms may be incorporated alongside arousal level evaluations. In addition, the clinical use of ICU‐specific assessment tools tailored to the ICU environment can be further strengthened. For example, the Chelsea Critical Care Physical Assessment Tool, FSS‐ICU, and ICU Mobility Scale are recommended as suitable tools for evaluating rehabilitation performance in the ICU [41, 42]. Moreover, the use of activity measures more appropriate for acute ICU patients than the MBI could expand the scope of OT and ADL interventions delivered in this setting. Furthermore, this approach highlights the need for the development and validation of ICU‐specific OT outcome measures that more accurately capture OT‐related functional changes in critically ill patients.

A total of 656 ICU OT sessions were conducted in 158 patients, and only one adverse event (0.6%) was observed, confirming that ICU OT is feasible and safe in the ICU setting. This aligns with prior evidence on the safety of early mobilization and rehabilitation [43–45]. Although early mobilization and physical therapy–based rehabilitation have shown functional benefits [37, 46] and ICU rehabilitation is generally considered safe [32, 43], evidence specific to OT remains limited. A recent review identified only nine adult ICU OT studies from 2012 to 2021, highlighting the lack of OT‐focused research, standardized protocols, and established feasibility [47]. In the context of this limited evidence, this study provides foundational evidence to support future research on the feasibility of ICU OT interventions in routine clinical practice.

A comparison group was not included because this study was designed as an initial feasibility assessment of ICU OT implementation in a real‐world clinical setting. This study design was based on the following considerations. First, at the time of the study, no globally established or standardized ICU‐specific OT protocol existed, making it difficult to define a consistent “standard care” control group for comparison. Second, given the retrospective nature of this study, the analysis was based on existing medical records of patients who had already received OT according to institutional clinical practice, and it was therefore not feasible to retrospectively assign patients to a nonintervention group. Accordingly, instead of direct comparison with a control group, the findings were interpreted based on absolute implementation metrics derived from the program. Feasibility indicators, including the proportion of patients meeting the eligibility criteria among those referred for ICU rehabilitation, program interruption rate, time to initiation of OT after prescription, missed or deferred sessions and their reasons, and adherence to the planned ICU OT program, were quantitatively described, with additional consideration of potential clinical factors influencing these outcomes. This approach allowed us to evaluate the feasibility and safety of program implementation in a real‐world clinical setting. Therefore, the findings should be interpreted descriptively, focusing on implementation metrics rather than comparative effectiveness, and may serve as a foundation for future prospective randomized controlled trials.

Other limitations of the study should also be considered when interpreting the findings of this study. First, ICU OT was delivered by a single therapist, reflecting limited human resources. This constraint reduced the number of treatment sessions, limited generalizability, and restricted opportunities for repeated assessments. Consequently, changes in functional outcomes over time could not be adequately evaluated, and the effectiveness of the intervention could not be determined. Second, practical constraints related to the ICU environment and limited treatment time restricted the use of additional patient‐reported outcome measures, particularly questionnaire‐based assessments. Third, because the timing of transfer from the ICU to the general ward is often unpredictable, standardized reassessments at ICU discharge or at the end of the ICU OT program could not be consistently performed. In addition, the absence of a structured follow‐up protocol limited the evaluation of post‐ICU outcomes. Fourth, patients who were likely to refuse therapy, such as those with severe pain, fatigue, anxiety, delirium, or poor cooperation, were largely excluded during the initial eligibility screening. As a result, the feasibility and acceptability observed in this study may have been overestimated compared with routine clinical practice. Finally, although patient heterogeneity was observed, this study was not designed to assess subgroup differences, and subgroup analyses were not conducted because the sample size was not sufficient to support adequately powered or reliable comparisons. Future studies with larger sample sizes should explore potential differences across diagnostic or functional subgroups.

In future studies, several directions should be considered to strengthen the evidence base for ICU OT and to expand its clinical applicability. First, implementing specific therapy doses and intervention strategies should be considered to broaden access for a wider range of ICU patients. Second, larger scale randomized controlled trials incorporating standardized reassessment time points and structured follow‐up assessment protocols are needed to evaluate a wide range of clinical outcomes, including functional improvement, patient‐centered outcomes, and follow‐up outcomes after ICU discharge. Third, to advance from feasibility studies toward clinical studies evaluating effectiveness, the development and validation of ICU‐specific OT outcome measures should be prioritized. Finally, improvements in education regarding ICU OT referral criteria and screening procedures may facilitate more appropriate identification of patients who require ICU OT in real‐world clinical settings and improve evaluation of the feasibility and acceptability of ICU OT programs, including the burden of treatment refusal.

### 4.1. Implications for OT

The individualized ICU OT program developed in this study provides foundational evidence supporting the feasibility and safety of implementing OT in ICU settings. In future clinical practice, securing sufficient OT resources and implementing individualized interventions based on patient characteristics, while considering therapy dose, may facilitate more stable and scalable delivery of ICU OT. Such approaches may further support the integration of individualized OT into routine ICU care while maintaining feasibility and safety.

## 5. Conclusions

We demonstrate the feasibility and safety of implementing individualized ICU OT programs that include early mobility, cognitive interventions, and upper extremity function training. Using predefined eligibility criteria and session‐by‐session reassessment, ICU OT was delivered without significant safety‐related events. These findings provide foundational information for assessing the feasibility and safety of individualized ICU OT implementation in critical care settings.

**Nomenclature**
ICU: intensive care unit
OT: occupational therapy
RASS: Richmond Agitation–Sedation Scale
HGS: handgrip strength
K‐MMSE2: Korean Mini‐Mental State Examination‐2nd Edition
MMT: Manual Muscle Test
PROM: passive range of motion
AAROM: active‐assistive range of motion
ADLs: activities of daily living

## Author Contributions

Conceptualization: S.H.J. and S.E.H. Investigation and data curation: S.H.J. and C.H.L. Formal analysis: S.H.J. and S.E.H. Funding acquisition and project administration: Y.A.Y. Methodology: S.H.J. and S.E.H. Supervision: S.E.H. and Y.A.Y. Validation: S.H.J. and C.H.L. Visualization: S.H.J. Writing—original draft: S.H.J. and S.E.H. Writing—review and editing: all authors.

## Funding

This work was supported by a grant from the Research Year of Inje University in 2023 (Grant No. 20240001).

## Conflicts of Interest

The authors declare no conflicts of interest.

## Supporting information


**Supporting Information** Additional supporting information can be found online in the Supporting Information section. Supporting Information 1. Inclusion and exclusion criteria. (A) Inclusion and exclusion in a retrospective data analysis. (B) Inclusion and exclusion in the ICU OT program. Supporting Information 2. K‐MMSE2 copyright permission. Supporting Information 3. Functional assessment tools: Korean Mini‐Mental State Examination‐2nd Edition (K‐MMSE2), handgrip strength (HGS) (kilograms), Korean version of the Modified Barthel Index (K‐MBI), and Richmond Agitation–Sedation Scale (RASS).

## Data Availability

The data that support the findings of this study are available from the corresponding author upon reasonable request.
