# Supplementary material for: Occupational Therapy in the Intensive Care Unit: A Retrospective Cohort Study
Source: Occup Ther Int. 2026 May 18;2026:4043807. doi: 10.1155/oti/4043807 (PMC13181319; doi:10.1155/oti/4043807)
Supplement: Supplementary file 1 — Supporting Information Additional supporting information can be found online in the Supporting Information section. Supporting Information 1. Inclusion and exclusion criteria. (A) Inclusion and exclusion in a retrospective data analysis. (B) Inclusion and exclusion in the ICU OT program. Supporting Information 2. K‐MMSE2 copyright permission. Supporting Information 3. Functional assessment tools: Korean Mini‐Mental State Examination‐2nd Edition (K‐MMSE2), handgrip strength (HGS) (kilograms), Korean version of the Modified Barthel Index (K‐MBI), and Richmond Agitation–Sedation Scale (RASS). [file OTI-2026-4043807-s001.docx]

**Supplementary Materials**

**Supplementary Material 1. Inclusion and Exclusion Criteria.**

***A*. *Inclusion and Exclusion in a Retrospective Data Analysis***

Patients were included in the retrospective study if they were admitted to any intensive care unit (ICU) at the institution (medical, surgical, cardiopulmonary, emergency, or semi-ICU) between February 2021 and December 2023 and received a prescription for ICU occupational therapy (OT) from the Department of Rehabilitation Medicine.

Patients were excluded if OT was not initiated due to transfer, death, or medical deterioration before the initial evaluation, or if essential medical records required for data analysis were incomplete or missing. The IRB approved a waiver of informed consent because this study was retrospective in design.

***B. Inclusion and Exclusion in the ICU OT Program***

*Inclusion in the ICU OT Program*

The inclusion criteria for the ICU OT program were as follows: the ability to follow simple instructions under light sedation (not induced by neuromuscular blockers) during OT and to control and cope with delirium without agitation, anxiety, alcohol withdrawal symptoms, convulsions, or epilepsy. Active ICU OT was recommended if there was no plan for early discharge to the general ward within 2 days, especially for patients without physical or mental impairments and those capable of walking independently and living daily before admission to the ICU (Figure 1).

*Exclusion from the ICU OT Program*

The exclusion criteria for the ICU OT program were as follows: ICU rehabilitation was not prescribed when patients presented with systolic blood pressure (SBP) <90 mmHg, diastolic blood pressure (DBP) <50 mmHg, heart rate <40 or >130 beats/min, respiratory rate <5 or >40 breaths/min, progressive acute respiratory failure, or hemodynamic instability with an oxygen saturation level <88%. Additionally, ICU rehabilitation was not prescribed when patients presented with increased intracranial pressure, overt gastrointestinal bleeding, acute myocardial infarction, the need for additional medical or surgical procedures, agitation or anxiety requiring sedation within 30 min, or the presence of an airway device, given the potential for rapid clinical deterioration.

After ICU OT was initiated, therapists continuously monitored clinical warning signs corresponding to the exclusion criteria for the ICU OT program before and during each session to determine whether treatment should be discontinued. If clinical warning signs—such as exhibiting hemodynamic instability, ventilator incompatibility, unstable breathing, complaints of pain, or physical resistance—were observed, the session was immediately discontinued. After the session was discontinued, the physiological parameters corresponding to the exclusion criteria were promptly reassessed, and if the reassessment continued to meet the exclusion criteria for the ICU OT program, the OT prescription was terminated. All OT sessions discontinued according to these criteria were documented in the medical record and classified as adverse events. Adverse events were classified as OT-related when a temporal and physiological association with the OT intervention was clinically determined. Events that were clinically attributed to the natural course of underlying medical conditions or pre-existing hemodynamic instability, based on clinical assessment by the treating physician, were not classified as OT-related adverse events.

**Supplementary Material 2. K-MMSE2 copyright permission.**

[Provided as a separate file]

**Supplementary Material 3. Functional Assessment Tools**

***Korean-Mini–Mental State Examination-2^nd^ Edition (K-MMSE2)***

The K-MMSE2 is a revised version (2nd edition) of the MMSE, a simple cognitive screening tool developed by Folstein et al. (1975) to quantitatively assess cognitive dysfunction in elderly individuals and patients with brain injuries [1]. It is widely used worldwide and evaluates orientation to time, orientation to place, memory registration and recall, language abilities, and visuospatial construction abilities. Regarding test results, of the total 30 points, a score of ≤17 indicates severe cognitive decline, 18–23 indicates mild cognitive decline, and ≥24 indicates normal cognitive function. Regarding uneducated patients, 1 point was added to time orientation, 2 points to attention and calculation, and 1 point to language function to calculate the total score [2].

***Handgrip Strength (HGS) (kg)***

HGS was measured using a hand dynamometer (Model 78010; Lafayette Instrument Company, Lafayette, IN, USA) (the higher the value, the stronger the grip force) in a standardized posture established by the American Society of Hand Therapists [3]. In a sitting position, the shoulder joint was abducted, the elbow joint was positioned at 90°, the forearm remained neutral, and the wrist joint extended from 0° to 30°. Ulnar deviation was set between 0° and 15°. Both hands were tested thrice, and the measurements were averaged [3]. Low muscle strength was defined as HGS <28 kg for men and <18 kg for women [4,5]. HGS is correlated with cognitive and physical function in older adults [6].

***Korean version of the Modified Barthel Index (K-MBI)***

The K-MBI is an assessment tool for activities for daily living; it comprises 10 detailed items with a score range of 0–100 points and is a highly reliable and valid tool. The score distribution ranges from 0 to 5 for personal hygiene and bathing; 0 to 10 for feeding, toileting, stair climbing, dressing, bowel control, and bladder control; and 0 to 15 for ambulation and chair/bed transfer. If the patient uses a wheelchair more than performing usual walking, the score is measured with the wheelchair rather than the walking, and wheelchair mobility was scored from 0 to 5 points. Therefore, the maximal score for a patient using a wheelchair or bed for transfer in the ICU setting is 90 and thus cannot be 100. Scores of 0–24, 25–49, 50–74, 75–90, 91–99, and 100 indicate total, severe, moderate, mild, and minimal dependence, and complete independence, respectively [7].

***Richmond Agitation–Sedation Scale (RASS)***

The RASS is a commonly used tool in the ICU for evaluating the patient’s sedation-agitation level, with 0 indicating being alert; -1, drowsy; -5, unarousable; +1, restless; and +4, combative. Verbal stimuli, e.g., calling of names and opening of the eyes, and if there was no response, physical stimuli, e.g., shaking of the shoulders or rubbing of the sternum, were used to evaluate the degree of sedation [8]. The RASS can be used to repeatedly assess and detect rapid changes in sedation status [9].

**References**

[1] Folstein, M. F., Folstein, S. E., & McHugh, P. R. (1975). ‘Mini-Mental State’: A Practical Method for Grading the Cognitive State of Patients for the Clinician. *Journal of Psychiatric Research*, 12(3), 189–198. <https://doi.org/10.1016/0022-3956(75)90026-6>.

[2] Song, M., Lee, S. H., Yu, K. H., & Kang, Y. (2019). Development and Validation of the Full Version of Story Memory in the Korean-Mini Mental State Examination, 2nd Edition: Expanded Version (K-MMSE-2: EV). *Dementia and Neurocognitive Disorders*, 18(3), 96–104. <https://doi.org/10.12779/dnd.2019.18.3.96>.

[3] Liu, C. J., Marie, D., Fredrick, A., Bertram, J., Utley, K., & Fess, E. E. (2017). Predicting Hand Function in Older Adults: Evaluations of Grip Strength, Arm Curl Strength, and Manual Dexterity. *Aging Clinical and Experimental Research*, 29(4), 753–760. <https://doi.org/10.1007/s40520-016-0628-0>.

[4] Chen, L. K., Woo, J., Assantachai, P., Auyeung, T. W., Chou, M. Y., Iijima, K., Jang, H. C., Kang, L., Kim, M., Kim, S., Kojima, T., Kuzuya, M., Lee, J. S. W., Lee, S. Y., Lee, W. J., Lee, Y., Liang, C. K., Lim, J. Y., Lim, W. S., Peng, L. N., Sugimoto, K., Tanaka, T., Won, C. W., Yamada, M., Zhang, T., Akishita, M., & Arai, H. (2020). Asian Working Group for Sarcopenia: 2019 Consensus Update on Sarcopenia Diagnosis and Treatment. *Journal of the American Medical Directors Association*, 21(3), 300–307.e2. <https://doi.org/10.1016/j.jamda.2019.12.012>.

[5] Kim, M., & Won, C. W. (2020). Sarcopenia in Korean Community-Dwelling Adults Aged 70 Years and Older: Application of Screening and Diagnostic Tools from the Asian Working Group for Sarcopenia 2019 Update. *Journal of the American Medical Directors Association*, 21(6), 752–758. <https://doi.org/10.1016/j.jamda.2020.03.018>.

[6] Chen, K. K., Lee, S. Y., Pang, B. W. J., Lau, L. K., Jabbar, K. A., Seah, W. T., Tou, N. X., Yap, P. L. K., Ng, T. P., & Wee, S. L. (2022). Associations of Low Handgrip Strength and Hand Laterality with Cognitive Function and Functional Mobility—The Yishun Study. *BMC Geriatrics*, 22(1), 677. <https://doi.org/10.1186/s12877-022-03363-2>.

[7] Jung, H. Y., Park, B. K., Shin, H. S., Kang, Y. K., Pyun, S. B., Paik, N. J., Kim, S. H., Kim, T. H., Han, T. R. (2007). Development of the Korean Version of Modified Barthel Index (K-MBI): Multi-center Study for Subjects with Stroke. *Journal of the Korean Academy of Rehabilitation Medicine*, 31(3), 283–297.

[8] Sessler, C. N., Gosnell, M. S., Grap, M. J., Brophy, G. M., O’Neal, P. V., Keane, K. A., Tesoro, E. P., & Elswick, R. K. (2002). The Richmond Agitation-Sedation Scale: Validity and Reliability in Adult Intensive Care Unit Patients. *American Journal of Respiratory and Critical Care Medicine*, 166(10), 1338–1344. <https://doi.org/10.1164/rccm.2107138>.

[9] Ely, E. W., Truman, B., Shintani, A., Thomason, J. W. W., Wheeler, A. P., Gordon, S., Francis, J., Speroff, T., Gautam, S., Margolin, R., Sessler, C. N., Dittus, R. S., & Bernard, G. R. (2003). Monitoring Sedation Status over Time in ICU Patients: Reliability and Validity of the Richmond Agitation-Sedation Scale (RASS). *JAMA*, 289(22), 2983–2991. <https://doi.org/10.1001/jama.289.22.2983>.
